# Biological Exposure Indices of Pyrrole Adducts in Serum and Urine for Hazard Assessment of *n*-Hexane Exposure

**DOI:** 10.1371/journal.pone.0086108

**Published:** 2014-01-22

**Authors:** Hongyin Yin, Chunling Zhang, Ying Guo, Xiaoying Shao, Tao Zeng, Xiulan Zhao, Keqin Xie

**Affiliations:** 1 Institute of Toxicology, School of Public Health, Shandong University, Jinan, Shandong Province, China; 2 Jinan Municipal Center for Disease Control & Prevention, Jinan, Shandong Province, China; 3 Nutrition Department, Shandong Jiaotong Hospital, Jinan, Shandong Province, China; The Ohio State University, United States of America

## Abstract

**Background:**

Pyrrole adducts might be used as a biomarker for monitoring occupational exposure to *n*-hexane, but the Biological Exposure Indices of pyrrole adducts in serum and urine are still unknown. The current study was designed to investigate the biological exposure limit of pyrrole adducts for hazard assessment of *n*-hexane.

**Methods:**

Male Wistar rats were given daily dose of 500, 1000, 1500, 2000, 4000 mg/kg bw *n*-hexane by gavage for 24 weeks. The levels of pyrrole adducts in serum and urine were determined at 8, 24 hours postdose once a week. The Biological Exposure Indices was evaluated by neurological evaluation and the levels of pyrrole adducts. The difference in pyrrole adducts formation between humans and rats were estimated by using *in vitro* test.

**Results:**

Dose-dependent effects were observed between the doses of *n*-hexane and pyrrole adducts in serum and urine, and the levels of pyrrole adduct in serum and urine approached a plateau at week 4. There was a significantly negative correlation between the time to paralysis and the level of pyrrole adducts in serum and urine, while a positive correlation between gait score and levels of pyrrole adducts in serum and urine was observed. *In vitro*, pyrrole adducts formed in human serum was about two times more than those in rat serum at the same level of 2,5-HD.

**Conclusion:**

It was concluded that the BEIs of pyrrole adducts in humans were 23.1±5.91 nmol/ml in serum 8 h postdose, 11.7±2.64 nmol/ml in serum 24 h postdose, 253.8±36.3 nmol/ml in urine 8 h postdose and 54.6±15.42 nmol/ml in urine 24 h postdose.

## Introduction

**Figure 1:**
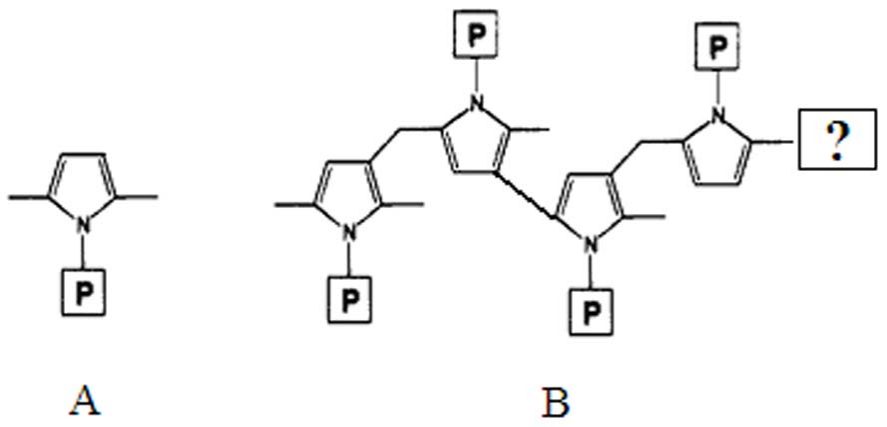
The structure of the pyrrole adduct. A: 2,5-dimethylpyrrole adducts, B: cross-linked pyrrole adducts.

Occupational exposure to *n*-hexane could produce nerve damage classified as central-peripheral distal axonopathy [1], [2]. Previous studies have demonstrated that 2,5-hexanedione (2,5-HD) was a major toxic metabolite which could react with the lysine ε-amino group and form pyrrole adducts (Figure 1A) [3]–[5], then yielding covalent cross-linking of pyrrolylated protein by secondary autoxidation reactions (Figure 1B) [6]–[8]. Previous studies have supported that the formation of pyrrole adducts is a critical step in γ-diketone neurotoxicity [9], [10].

As *n*-hexane is widely used in the world, it is urgent to estimate the exposure of *n*-hexane for the protection of workers. Ambient air and biological monitoring were frequently-used methods for hazard assessment. Therein, the biological monitoring might offer several advantages over ambient air monitoring in the evaluation of internal dose and the estimation of health risk [11]–[13]. Parent chemicals, metabolites and adducts formed with proteins or nucleic acids in biological media might be used for biological monitoring [14]–[18].

Free 2,5-HD (without hydrolysis) has been used as a biomarker for urinary biological monitoring of occupational exposure to *n*-hexane. The Biological Exposure Indices (BEIs) recommended by American Conference of Governmental Industrial Hygienists' (ACGIH) is 0.4 mg/L. However, the equipment for the determination of 2,5-HD is gas chromatography or gas chromatography mass spectrometry (GC, GC/MS), and the pretreatment of the urine sample is also complex. Therefore, the 2,5-HD was not an convenient biomarker [19]–[21].

As described above, 2,5-HD could react with the lysine ε-amino group and form 2,5-dimethylpyrrole ring. Although several studies have suggested that 2,5-dimethylpyrrole might be a biomarker for *n*-hexane, the correlation between pyrrole adducts and neuropathy was unclear, and the BEIs of pyrrole adducts was still unknown [22], [23]. The 2,5-dimethylpyrrole ring could be detected by colorimetric assay with 4-dimethylamino-benzaldheyde (DMBA) using an UV-Vis spectrophotometer which is easy to operate[24]. Levels of pyrrole adducts determined by DMBA assay might be underestimated due to the autooxidization of pyrrole adducts, the formation of stable secondary pyrrole adducts with SH groups and steric factors [10], [25]–[28]. However, the pyrrole adducts detected by DMBA method showed a good dose-dependent effect with *n*-hexane [23], [29], which suggests that this limitation could not hamper the use of pyrrole adducts as a sensitive biomarker for *n*-hexane exposures. Therefore, the total pyrrole adducts might be not necessary, which is similar to the fact that free 2,5-HD could be used as a biomaker instead of total 2,5-HD (ACGIH).

In order to evaluate the possibility of pyrrole adducts as a biomarker of *n*-hexane and determine the BEIs for hazard assessment, we designed the current study to investigate the levels of the pyrrole adducts in serum and urine of rats exposed to *n*-hexane and analyze the correlation between pyrrole adducts and neuropathy.

## Materials and Methods

### Materials

2,5-dimethylpyrrole, 2,5-hexaneketone (2,5-HD), 4-dimethylamin- obenzaldehyde and boron fluoride (14% solution) were purchased from Sigma-Aldrich, Inc. (St. Louis, MO, USA). Ethanol, cyclohexanone, dichloromethane were purchased from Hongyan chemical reagent factory (Tianjin, China). All chemicals were of the highest grade commercially available.

### Animals Experiments

#### Ethics Statement

The experiments were conducted in accordance with the NIH Guide for Care and Use of Laboratory Animals and the principles in the “Use of Animals in Toxicology”, and were approved by the Ethics Committee of Shandong University Institute of Preventive Medicine (Permit Number: 20130801).

#### Animals

Adult male Wistar rats (Experimental Animal Center of Shandong University), weighing 280–300 g, were used in this experiment. Animals had free access to tap water and standard rat chow. Animal housing and care followed currently accepted standards of the NIH Guide for Care and Use of Laboratory Animals and the principles in the “Use of Animals in Toxicology”. The animal room temperature was maintained at 20±2°C, with a relative humidity control of 50±10%, and 12 hours light/dark cycle.

#### Animals Treatments

After 7 days for acclimatization, the animals were randomly divided into six groups (n = 8). Experimental group rats were treated with *n*-hexane daily by gavage at a dosage of 500, 1000, 1500, 2000, 4000 mg/kg/day seven times per week for 24 weeks, respectively. The corresponding control group rats received an equivalent volume of 0.9% saline. Blood and urine samples were collected at 8, 24 hours postdose once a week. Blood was drawn from the inner canthus and the urine was obtained by the tenderness stimulation micturition. Serum was acquired by centrifugation at 2000 rpm for 10 min and then kept frozen at approximately −70°C until analysis.

Neurological testing was carried out along with the collection of blood and urine samples. To measure gait abnormalities, rats were placed in a clear plexiglass box and were observed for 3 min [30]–[32]. Following observation, a gait score was assigned from 1 to 4, where 1 = a normal, unaffected gait; 2 = a slightly affected gait (tip-toe walking, slight ataxia, and hindlimb weakness); 3 = a moderately affected gait (obvious movement abnormalities characterized by dropped hocks and tail dragging); and 4 = a severely affected gait (frank hindlimb weakness and an inability to rear). A trained, blinded observer who was not involved in animal care or *n*-hexane exposure performed the behavioral evaluation. Three successive measurements were averaged for each *n*-hexane-intoxicated or control rat [33].

### Collection of Normal Human Serum and Urine

One hundred adults, at age 20–50 years old without the habit of smoking and excessive drinking, were selected for the collection of morning serum and spot morning urine under the fasting conditions. The blood was drawn from ulnar vein and the urine was collected by urine glass. Serum was acquired by centrifugation at 2000 rpm for 10 min. Serum and urine samples were kept frozen at approximately -70°C until analysis. This study were conducted according to the Declaration of Helsinki, and approved by the Ethics Committee of Shandong University Institute of Preventive Medicine (Permit Number: 20131001), and all participants provided a written informed consent in this study.

### Formation of Pyrrole Adducts *in vitro*

Human serum was obtained from from healthy adults as described above and rat serum was obtained from control group rats. Both were exposed to 2,5-HD (10, 50 µg/ml) at 37°C. The levels of pyrrole adducts formed in serum were detected at 0, 2, 4 and 10 hours after exposure.

### Pyrrole Adducts Detection

The pyrrole adducts were measured spectrophotometrically after reaction of 0.1 ml of urine or serum with 0.1 ml guanidine hydrochloride (70%) and 0.1 ml of Ehrlich's reagent (3% 4-dimethylamino- benzaldheyde in 40 ml of methanolic 14% boron trifluoride and 60 ml of ethanol) [24]. Absorption values were measured at 526 nm, using automatic microplate reader (Infinite® 200 PRO, TECAN Inc. Switzer). The calculations were based on standard curve prepared with different concentrations of 2,5-dimethylpyrrole and the values were expressed as nmol/ml [7].

### Statistical Analysis

All data were expressed as mean ± S.D. SPSS13.0 statistical software was used for statistical analysis. All data were analyzed using one-way analysis of variance (ANOVA), followed by Student-Newman-Keuls (SNK) test. The correlations were analyzed by linear regression. The differences were significant at *P*<0.05.

## Results

### Changes of Body Weight and Clinical Signs

**Figure 2:**
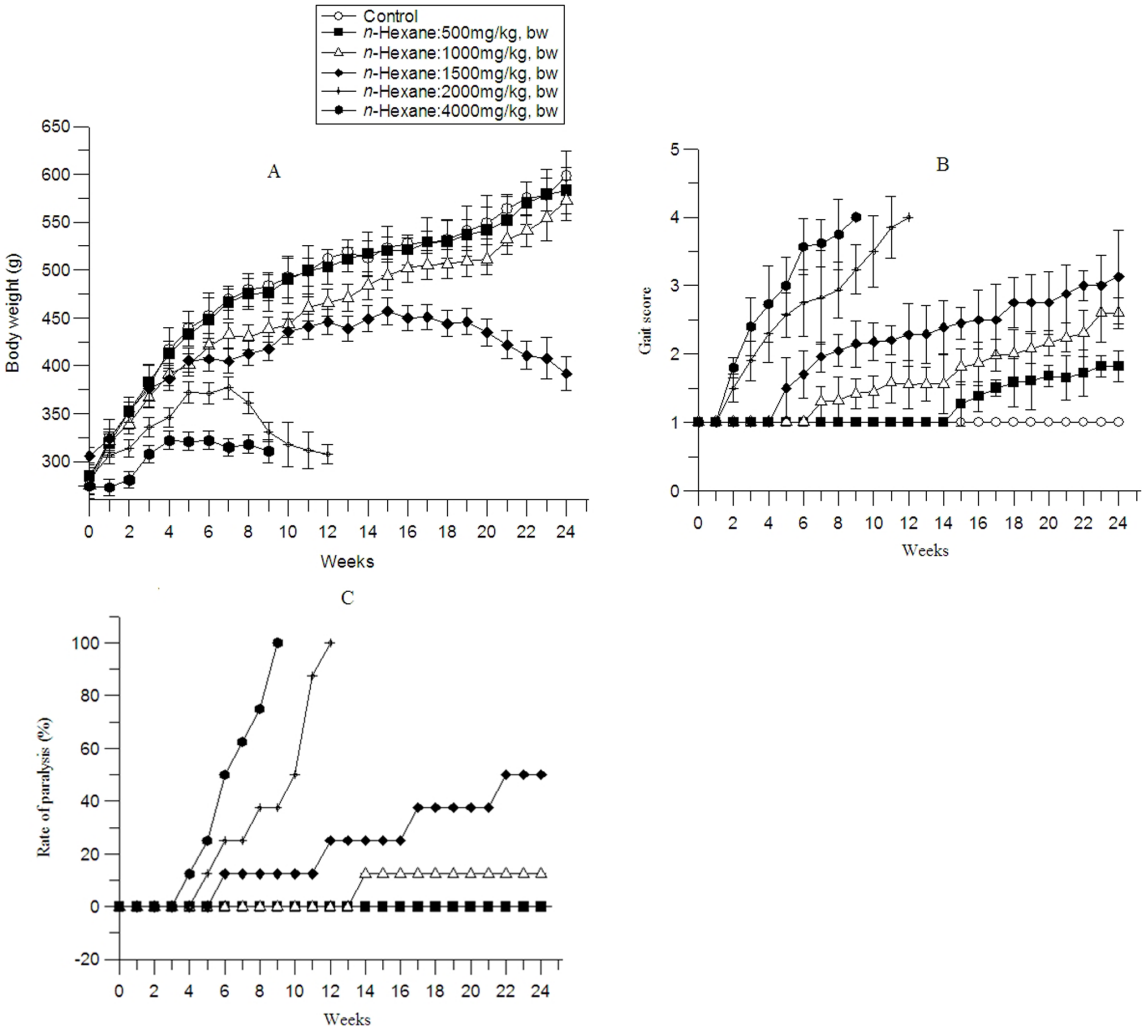
Effects of n-hexane on rats body weight, gait score and the rate of paralysis of rats. The data were expressed as mean±S.D. (n = 8).

Significant reductions of weight gain were observed in rats exposed to *n*-hexane (1000, 1500, 2000, 4000 mg/kg), while no reduction was observed in 500 mg/kg group. Body weights of rats at the exposure levels of 4000 mg/kg, 2000 mg/kg, 1500 mg/kg, 1000 mg/kg group showed significant differences from the control rats since week 1, 2,4 and 4 (*P*<0.05), respectively. As the intoxication continued, the mean weight gain slowed down gradually, and the body weights of rats in 1500 mg/kg, 2000 mg/kg, 4000 mg/kg groups began to decrease after 4, 7, 12 weeks, respectively. At the end of the experiment, the mean body weight of 4000 mg/kg, 2000 mg/kg, 1500 mg/kg, 1000 mg/kg groups were 64.4%, 60.1%, 73.7% and 95.8% of their age-matched controls, respectively (Figure 2 A).

At the beginning of *n*-hexane exposure, rats exhibited a normal, unaffected gait. Rats in 500, 1000, 1500, 2000, 4000 mg/kg *n*-hexane exposure groups began to show a progressive development of gait abnormalities from week 15, 7, 5, 2, 2, respectively. All rats in 1500, 2000, 4000 mg/kg groups developed paralysis in weeks 18, 12 and 9 respectively, 12.5% rats in 1000 mg/kg group got paralyzed at the end of the experiment and no paralysis was observed in 500 mg/kg group (Figure 2 B & C).

### Changes of Pyrrole Adducts Levels in Serum and Urine of Rats Exposed to *n*-Hexane for 24 Weeks

Pyrrole adducts levels in serum and urine sample were examined at 8 and 24 hours postdose once a week for 24 weeks to investigate the changes of the pyrrole adducts. Throughout the experiment, significant differences in pyrrole adducts levels in serum and urine were observed among *n*-hexane-treated groups and control group (*P*<0.05).

**Figure 3:**
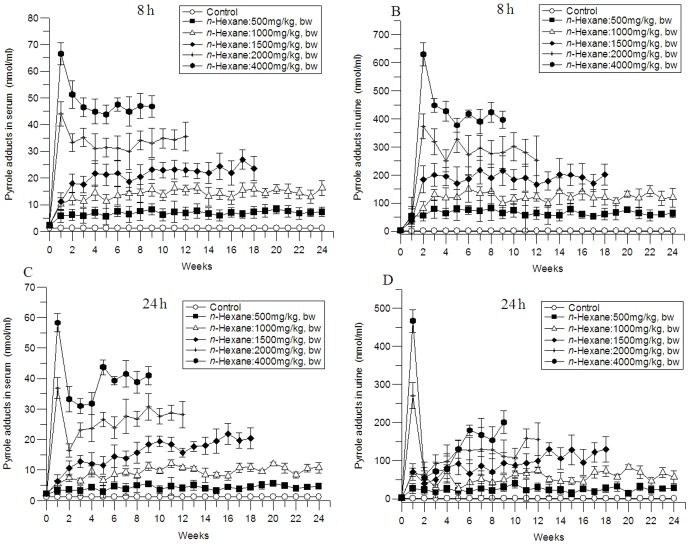
Changes of pyrrole adducts levels in serum and urine of rats exposed to *n*-hexane for 24 weeks. The data were expressed as mean ± S.D. (n = 8). A: pyrrole adducts in serum 8 hours postdose; B: pyrrole adducts in urine 8 hours postdose; C: pyrrole adducts in serum 24 hours postdose; D: pyrrole adducts in urine 24 hours postdose.

As shown in Figure 3, dose-dependent effects of *n*-hexane on pyrrole adducts levels in serum and urine were observed. The levels of Pyrrole adducts in serum and urine of rats exposed to 4000 and 2000 mg/kg *n*-hexane reached peak levels at week 1, followed by a decline lasting for about 3 weeks, approaching a plateau level at week 4. At 1500, 1000 and 500 mg/kg doses, no peak level was observed, levels of pyrrole adduct increased with time, and approached a plateau level at week 3.

### Correlations between the Time to Paralysis and the Pyrrole Adducts Levels in Serum and Urine at the Time of Paralysis Onset

**Figure 4:**
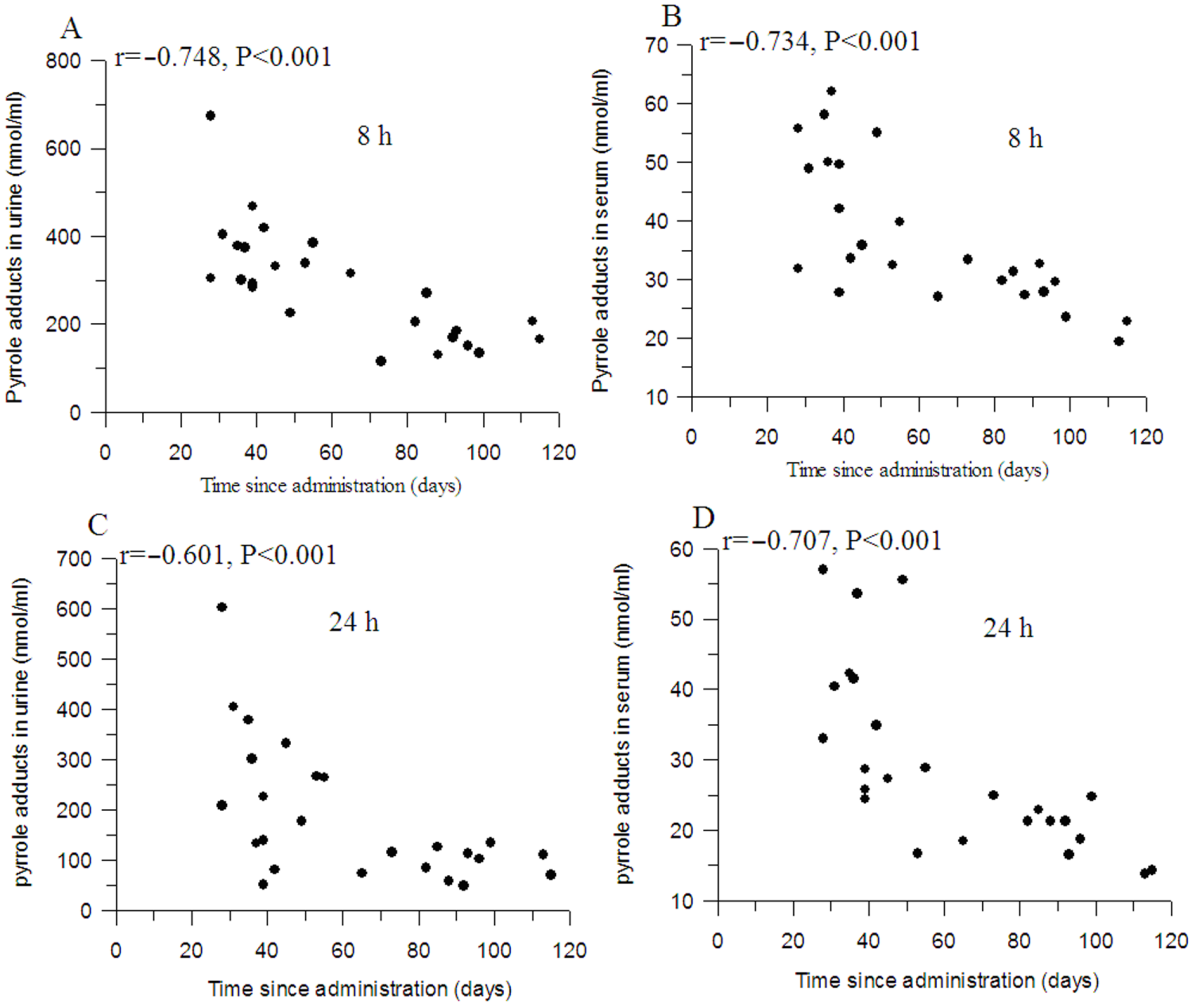
Correlations between the time to paralysis and the pyrrole adducts levels in serum and urine at time of paralysis. A: pyrrole adducts in serum 8 hours postdose; B: pyrrole adducts in urine 8 hours postdose; C: pyrrole adducts in serum 24 hours postdose; D: pyrrole adducts in urine 24 hours postdose.

At the end of the experiment, paralysis was evident in 25 rats. However, the time to paralysis was different for each rat. In order to investigate the contribution of pyrrole adducts to *n*-hexane-induced neuropathy, the relationships between the time to paralysis and pyrrole adducts levels in serum and urine were examined by correlation analysis. As shown in Figure 4, significantly negative correlations were observed between the time to paralysis and the pyrrole adducts levels in serum 8 h postdose (r = −0.734, *P*<0.001), in serum 24 h postdose (r = −0.707, *P*<0.001), in urine 8 h postdose (r = −0.748, *P*<0.001) and in urine 24 h postdose (r = −0.601, *P*<0.001).

### Correlations between the Gait Score and the Pyrrole Adducts Levels in Serum and Urine at Week 8

**Figure 5:**
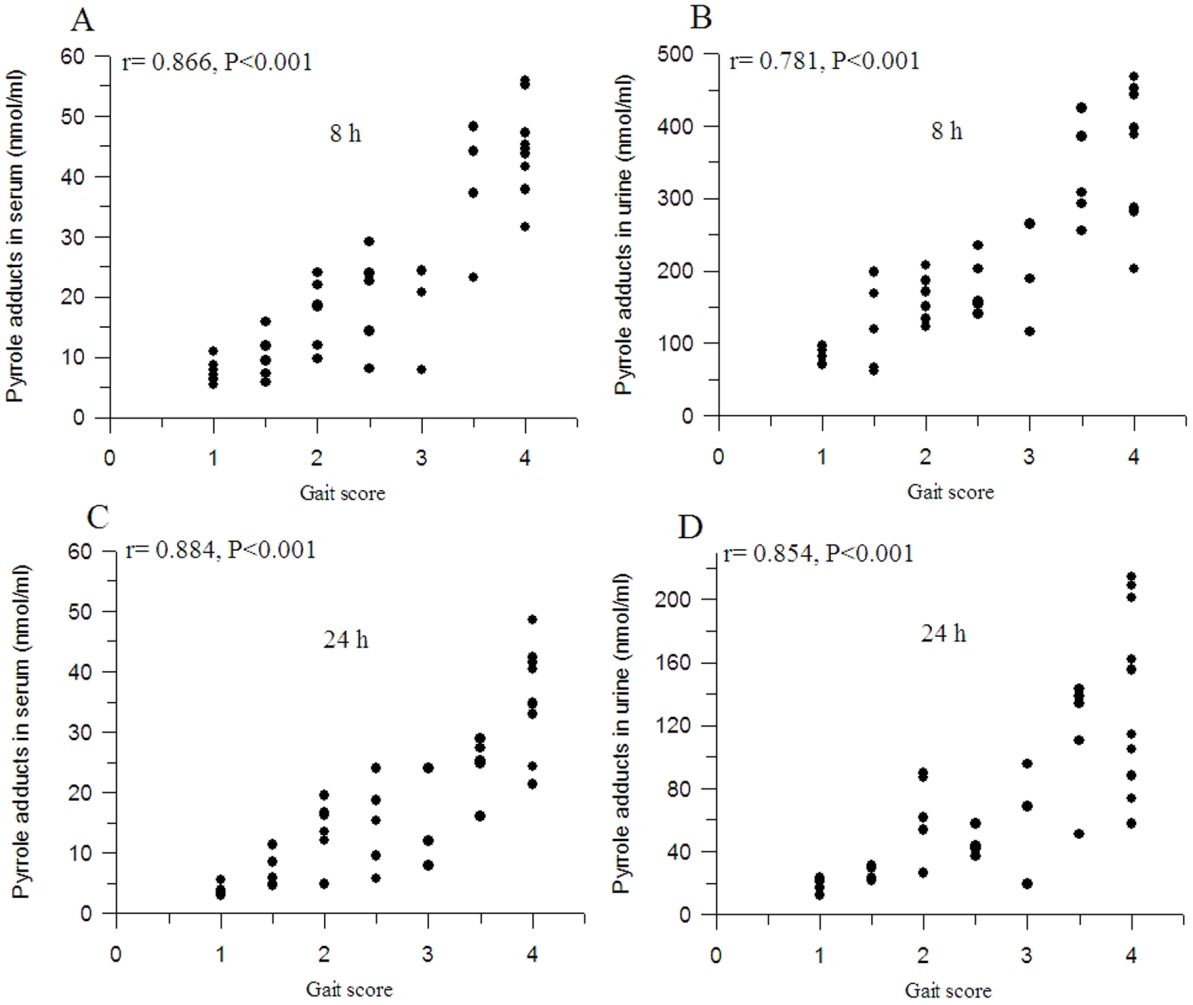
Correlations between the gait score and the pyrrole adducts levels in serum and urine in week 8. A: pyrrole adducts in serum 8 hours postdose; B: pyrrole adducts in urine 8 hours postdose; C: pyrrole adducts in serum 24 hours postdose; D: pyrrole adducts in urine24 hours postdose.

At week 8, different gait scores were observed for experimental animals. The relationships between the gait score and the pyrrole adducts levels in serum and urine were examined by correlation analysis to investigate the contribution of pyrrole adducts to *n*-hexane-induced neuropathy. As shown in Figure 5, significant correlations were noted between the gait score and the pyrrole adducts concentrations in serum 8 h postdose (r = 0.866, *P*<0.001), in serum 24 h postdose (r = −0.884, *P*<0.001), in urine 8 h postdose (r = −0.781, *P*<0.001) and in urine 24 h postdose (r = −0.854, *P*<0.001)

### Biological Exposure Indices of Pyrrole Adducts in Rats Exposed to *n*-Hexane

In order to prevent and control the neuropathy induced by *n*-hexane, it was critical to obtain the Biological Exposure Indices of pyrrole adducts in serum or urine. As shown in Table 1, paralysis was observed in all experimental rats above the 1500 mg/kg dose level of *n*-hexane from weeks 9 to 18. At 1000 mg/kg dose level, only 12.5% rats developed paralysis symptom after exposure to *n*-hexane for 24 weeks. However, no paralysis symptom was observed in animals exposed at 500 mg/kg dose level, and the corresponding levels of the pyrrole adducts in serum and urine at 8 hour and 24 hour were 6.90±0.75, 65.08±9.31, 4.24±0.75 and 24.37±5.95 nmol/ml.

**Table 1:** Effects of different dosages of *n*-hexane on paralysis rate and pyrrole adducts concentrations in serum and urine.

Group (*n*-hexane)	Paralysis rate (%)	Endpoint time* (weeks)	pyrrole adducts in serum (nmol/ml) **		pyrrole adducts in urine (nmol/ml) **	
			8 h	24 h	8 h	24 h
Control	0%	24	1.4±0.15	1.3±0.12	0.81±0.32	0.78±0.23
**500 mg/kg**	**0%**	**24**	**6.90**±**0.75**	**4.24**±**0.75**	**65.08**±**9.31**	**24.37**±**5.95**
1000 mg/kg	12.5%	24	14.09±1.69	9.31±1.78	118.1±23.12	56.50±14.77
1500 mg/kg	100%	18	21.34±3.40	15.78±4.01	182.2±39.1	91.07±26.17
2000 mg/kg	100%	12	33.99±3.65	26.67±4.88	293.4±35.8	117.9±28.62
4000 mg/kg	100%	9	48.80±6.97	39.86±8.26	438.9±80.4	149.4±55.19

*Time when all rats were observed hind-limb paralysis or the experiment stopped

*Average levels of pyrrole adducts in whole experiments.

Gait score and its corresponding pyrrole adducts levels in serum and urine of rats at week 8 were shown in Table 2. As mentioned above, gait score 1 means normal, unaffected gait which was also the basic aim of the control of the neuropathy. Therefore, the corresponding pyrrole adducts levels in serum and urine of gait score 1 might be considered as the BEIs, which were 7.7±1.97 nmol/ml in serum 8 h postdose, 3.9±0.88 nmol/ml in serum 24 h postdose, 84.6±12.1 nmol/ml in urine 8 h postdose and 18.2±5.14 nmol/ml in urine 24 h postdose.

**Table 2:** Gait score and its corresponding pyrrole adducts levels in serum and urine of rats at week 8.

Gait score	Number of rats	Time (weeks)	pyrrole adducts in serum (nmol/ml) *		pyrrole adducts in urine (nmol/ml) *	
			8 h	24 h	8 h	24 h
Control	8	8	1.4±0.15	1.3±0.12	0.81±0.32	0.78±0.23
**1.0**	**6**	**8**	**7.7**±**1.97**	**3.9**±**0.88**	**84.6**±**12.1**	**18.2**±**5.14**
1.5	5	8	10.1±3.97	7.0±2.85	123.0±60.7	25.6±4.24
2.0	6	8	17.4±5.58	13.8±5.12	162.2±32.2	57.4±27.9
2.5	5	8	18.4±6.97	14.6±7.25	182.2±47.3	47.4±9.63
3.0	3	8	19.3±10.73	14.6±8.39	180.2±60.1	61.1±38.76
3.5	5	8	38.2±9.08	24.5±5.02	335.2±66.9	115.6±38.26
4.0	10	8	45.0±7.28	36.3±8.52	365.5±93.9	138.1±58.2

*Average levels of pyrrole adducts in the same levels of gait score.

### Estimation of the BEIs of Pyrrole Adducts in Human Serum or Urine

The pyrrole adducts levels in serum and urine of humans, who were not exposed to *n*-hexane were determined. As shown in Table 3 **,** the pyrrole adducts levels in serum and urine of normal humans, were rather low, having no significant difference with the rats of control group. Besides, there was no difference among different age and gender groups.

**Table 3:** Levels of pyrrole adducts in serum and urine of normal humans.

Age	Gender	Number of the sample	Pyrrole adducts in serum (nmol/ml)	Pyrrole adducts in urine (nmol/ml)
21–30	Male	12	1.47±0.38	0.68±0.39
	Female	15	1.53±0.63	0.79±0.43
31–40	Male	16	1.35±0.33	0.78±0.54
	Female	15	1.41±0.26	0.68±0.35
41–50	Male	22	1.55±0.63	0.87±0.57
	Female	20	1.62±0.49	0.76±0.54

**Figure 6:**
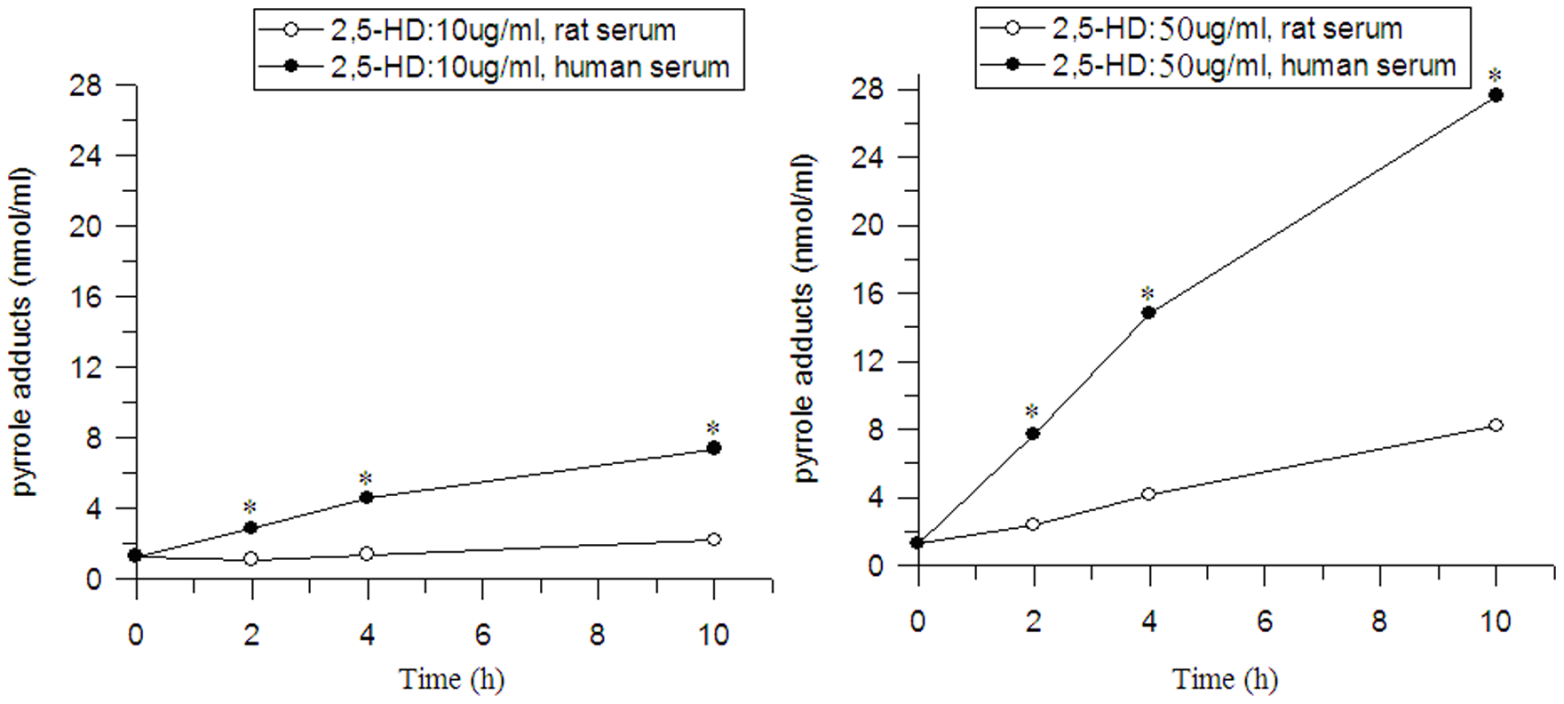
Difference in pyrrole adducts formation between the human serum and rat serum. *P<0.05 versus human serum.

An *in vitro* study was carried out to discuss the difference in pyrrole adducts formation between the human serum and rat serum for estimation of the BEIs of pyrrole adducts in human serum and urine. As shown in Figure 6, the exposure of rat and human serum to 10, 50 µg/ml 2,5-HD caused a dose and time dependent increase in the pyrrole adducts levels. However, the pyrrole adducts formed in human serum was about two times more than that in rat serum at the same level of 2,5-HD. Considering this relationship, therefore, the BEIs of pyrrole adducts for humans were 23.1±5.91 nmol/ml in serum 8 h postdose, 11.7±2.64 nmol/ml in serum 24 h postdose, 253.8±36.3 nmol/ml in urine 8 h postdose and 54.6±15.42 nmol/ml in urine 24 h postdose.

## Discussion

Occupational health maintenance requires several approaches to prevent health impairment that might result from excessive exposure to chemicals. Early detection of hazardous exposure might significantly decrease impairment, by controlling the level of exposure through appropriate preventive measures. The ambient air and biological monitoring were the routine assessment for the detection of possible health risks. This assessment requires the definition of permissible levels of exposure. However, the biological monitoring might offer several advantages over environmental monitoring to evaluate the internal dose and to estimate the health risk.

*n*-Hexane is a solvent that has many uses in the chemical and food industries, either in pure form or as a component of the mixture commercial hexane. Exposure to *n*-hexane would produce peripheral neuropathy [1], [2]. Although the urinary 2,5-HD has been considered as a biological monitoring biomarker for preventing the adverse effects induced by *n*-hexane, some points should be addressed with regard to the interpretation of the results of biological monitoring when this indicator is used[19], [21], [34], [35]. The method for the determination of 2,5-HD and the co-exposure to methyl ethyl ketone both affect the urinary 2,5-HD as a useful indicator[36]–[38]. The ideal biologic marker for risk assessment purposes should be a quantitative measurement of a chemical, biochemical, and functional or morphological change in the system that is initiated by a chemical and which results in pathologic change and overt toxicity [39]. Informative biomarkers detectable in serum or urine are convenient for researchers and clinicians since blood or urine is routinely and easily obtained [40], [41].

It has been suggested by many researches that pyrrole adducts might serve as a biomarker [22], [23], [42]. However, the correlation between the neuropathy and the pyrrole adducts levels was still unclear. In the present study, the changes of pyrrole adducts levels in serum and urine of rats exposed to different dosages of *n*-hexane for 24 weeks were studied. The results showed that rats displayed different severities of neuropathy after exposure to different dosages of *n*-hexane. A time and dose dependent effect of *n*-hexane on pyrrole adducts levels in serum and urine was observed. There were significantly positive correlations between the gait score and the pyrrole adducts levels in serum and urine, and negative correlations between the time to paralysis and the pyrrole adducts concentrations in serum and urine. All of these results suggested that the pyrrole adducts in serum or urine might serve as a biomarker for *n*-hexane exposure.

The BEIs are the reference values for the evaluation of potential health hazards in occupational hygiene. The BEIs is based upon each chemical having a reasonably safe level of exposure below which significant illness, injury, or discomfort will seldom happen [43]–[46]. It has been documented that the main damage caused by *n*-hexane was the peripheral neuropathy and the typical clinical manifestation was the muscle weakness or paralysis. Thus, the BEIs of pyrrole adducts were on the basis of the degree of gait abnormalities. In the present study, the rats exposed to *n*-hexane were all or partly paralyzed above the 1000 mg/kg dose level of *n*-hexane from week 9 to week 24. No rat was paralyzed at the 5 00 mg/kg dose level in the end of the experiment. Thus, there was no risk of paralysis at its corresponding levels of pyrrole adducts. As mentioned above, the pyrrole adducts concentrations were also significantly positively correlated with the gait abnormality, which means the higher the level of pyrrole adducts, the more serious of the neuropathy induced by *n*-hexane. The gait score was one of the methods for the evaluation of neuropathy and a score of 1 means normal, unaffected gait which was also the basic aim of the control of the neuropathy. Therefore, its corresponding pyrrole adducts levels in serum and urine might be considered as the BEIs for rats.

In order to obtain the BEIs of pyrrole adducts in humans, the difference in the pyrrole adducts formation between the rat serum and human serum was compared. The results showed that pyrrole adducts formed in human serum was about two times more than that in rat serum at the same level of 2,5-HD. Therefore, it was assumed that the pyrrole adducts formed in human body would be about two times more than that in rat body when exposed to the same dose of 2,5-HD. It was concluded that the BEIs of pyrrole adducts in humans were 23.1±5.91 nmol/ml in serum 8 h postdose, 11.7±2.64 nmol/ml in serum 24 h postdose, 253.8±36.3 nmol/ml in urine 8 h postdose and 54.6±15.42 nmol/ml in urine 24 h postdose.

In summary, the current study demonstrated that the pyrrole adducts levels in serum and urine might serve as a biomarker for *n*-hexane exposure and proposed the BEIs of pyrrole adducts in humans. However, the BEIs in humans obtained in this study were concluded from the results of rats, more research is needed to determine whether or not the conclusion was suitable for the biological monitoring application.
